# Hemochromatosis Enhances Tumor Progression *via* Upregulation of Intracellular Iron in Head and Neck Cancer

**DOI:** 10.1371/journal.pone.0074075

**Published:** 2013-08-26

**Authors:** Michelle Lenarduzzi, Angela B. Y. Hui, Shijun Yue, Emma Ito, Wei Shi, Justin Williams, Jeff Bruce, Noriko Sakemura-Nakatsugawa, Wei Xu, Aaron Schimmer, Fei-Fei Liu

**Affiliations:** 1 Ontario Cancer Institute, University Health Network, Toronto, Ontario, Canada; 2 Department of Medical Biophysics, University of Toronto, Toronto, Ontario, Canada; 3 Radiation Medicine Program, University Health Network, Toronto, Ontario, Canada; 4 Department of Radiation Oncology, University of Toronto, Toronto, Ontario, Canada; 5 Dalla Lana School of Public Health, University of Toronto, Toronto, Ontario, Canada; University of South Alabama Mitchell Cancer Institute, United States of America

## Abstract

**Introduction:**

Despite improvements in treatment strategies for head and neck squamous cell carcinoma (HNSCC), outcomes have not significantly improved; highlighting the importance of identifying novel therapeutic approaches to target this disease. To address this challenge, we proceeded to evaluate the role of iron in HNSCC.

**Experimental Design:**

Expression levels of iron-related genes were evaluated in HNSCC cell lines using quantitative RT-PCR. Cellular phenotypic effects were assessed using viability (MTS), clonogenic survival, BrdU, and tumor formation assays. The prognostic significance of iron-related proteins was determined using immunohistochemistry.

**Results:**

In a panel of HNSCC cell lines, hemochromatosis (HFE) was one of the most overexpressed genes involved in iron regulation. *In vitro* knockdown of HFE in HNSCC cell lines significantly decreased hepcidin (HAMP) expression and intracellular iron level. This in turn, resulted in a significant decrease in HNSCC cell viability, clonogenicity, DNA synthesis, and Wnt signalling. These cellular changes were reversed by re-introducing iron back into HNSCC cells after HFE knockdown, indicating that iron was mediating this phenotype. Concordantly, treating HNSCC cells with an iron chelator, ciclopirox olamine (CPX), significantly reduced viability and clonogenic survival. Finally, patients with high HFE expression experienced a reduced survival compared to patients with low HFE expression.

**Conclusions:**

Our data identify HFE as potentially novel prognostic marker in HNSCC that promotes tumour progression *via* HAMP and elevated intracellular iron levels, leading to increased cellular proliferation and tumour formation. Hence, these findings suggest that iron chelators might have a therapeutic role in HNSCC management.

## Introduction

Head and neck squamous cell carcinoma (HNSCC) is the 6th most common cancer worldwide, with ~650,000 new cases diagnosed and ~350,000 deaths annually [1,2]. The majority of patients present with locally-advanced disease, and despite new treatment approaches, the 5-year disease free survival rates have stagnated at 30-40% [3]. These poor outcomes highlight the importance of developing novel therapeutic strategies to target this disease.

Iron is an essential element involved in multiple key processes including DNA and heme synthesis, Wnt signalling, and cellular metabolism [4,5]. Many cancer cells exhibit an increased demand for iron in order to maintain their high cellular turnover and DNA synthesis. Consequently, genes involved in iron regulation are often deregulated in cancers. Here, we report the overexpression of hemochromatosis (HFE) in HNSCC, and demonstrate its ability to alter intracellular iron and increase cell proliferation.

Hemochromatosis is trans-membrane glycoprotein, similar to the major histocompatibility class 1 type molecule (MHC) that associates with B_2_-microglobulin for intracellular transport to the plasma membrane [6]. At the membrane, HFE can bind to either transferrin receptor 1 (TRF1) or transferrin receptor 2 (TFR2). The binding sites of HFE and iron bound transferrin overlap at TFR1 [7], thereby regulating iron uptake into cells. However, more recent evidence also suggests that a central role of HFE is to stimulate the expression of the iron regulatory hormone, hepcidin (HAMP), either through binding with TFR2 [8], or independently [9]. The function of HAMP is to internalize and degrade ferroportin (FPN), the only cellular exporter of iron [10]; leading to an increase in intracellular iron levels by inhibiting iron release [10]. As an example, overexpression of HFE in macrophages and colon adenocarcinoma cell lines inhibited the efflux of cellular iron [11,12].

On the other hand, genetic mutations in *HFE* lead to the iron overload condition, hereditary hemochromatosis (HH). The most common form of HH is caused by a single base pair mutation in HFE which results in a C282Y substitution [6], which disrupts the interaction between HFE with B_2_-microglobulin, and therefore the guidance of HFE to the cell membrane. Patients with HH have inappropriately low levels of HAMP [8], highlighting the importance of HFE for HAMP. Low HAMP results in the release of iron from reticuloendothelial macrophages, and allows for the continuous absorption of iron from the gut, leading to excess iron in circulation [13]. Consequently, circulating transferrin becomes saturated, resulting in the accumulation of iron in the parenchymal cells of various end-organs, resulting in cirrhosis, diabetes, cardiomyopathy, and cancer [13,14].

In this present study, we report the over expression of HFE in HSNCC, which in turn increased cellular levels of HAMP, and intracellular iron. By perturbing intracellular iron levels, HFE can promote cell proliferation, DNA synthesis, Wnt signalling, and tumour formation*.*

## Materials and Methods

### Ethics Statement

All animal experiments were conducted in accordance to guidelines of the Animal Care Committee at the University Health Network (Toronto, Canada). The protocol was approved by the Animal Care Committee at the University Health Network (Protocol Number: 342.19).

Patient samples were collected from 26 HNSCC patients, with approval from the University Health Network Institutional Research Ethics Board, (REB approval # 07-0521-CE). These specimens included matched group of 12 relapsed and 14 non-relapsed patients, with a median follow-up time of 3 years. The clinical details on these 26 patients are provided in Table S1. Written or Oral consent could not be obtained from the patients due to the period of our cohort (2003-2007). Therefore, our University Health Network Institutional Research Ethics Board waived the requirement for written informed consent from the participants of this study.

### Cells Lines and Reagents

The human hypopharyngeal HNSCC FaDu cell line was obtained from the American Type Culture Collection (Manassas, VA), and cultured according to the manufacturer’s specifications. The human laryngeal squamous cell lines, UTSCC-8 and UTSCC-42a (kind gifts from R Grénman, Turku University Hospital, Turku, Finland) [15,16] were maintained with DMEM supplemented with 10% fetal bovine serum (Wisent, Inc) and 100 mg/L penicillin/streptomycin. The normal oral epithelial cells (NOEs) were purchased commercially and cultured in the recommended medium (Celprogen). All cells were maintained in a 37°C incubator with humidified 5% CO_2_, authenticated at the Centre for Applied Genomics (Hospital for Sick Children, Toronto, Canada) using the AmpF/STR Identifier PCR Amplification Kit (Applied Biosystems), and routinely tested for mycoplasma contamination using the Mycoalert detection kit (Lonza Group Ltd).

### Quantification of mRNA

Total RNA was extracted from cell lines using the Total RNA purification kit (Norgen), then reverse transcribed using SuperScript II Reverse Transcriptase (Invitrogen Canada) according to specifications. Transcript levels of iron regulating genes: ferroportin (FPN), hepcidin (HAMP), hemochromatosis (HFE), transferrin receptor 1 (TFR1), ferritin heavy chain (FTH1), ferritin light chain (FTL) and mitochondria ferritin (FTMT) were assessed by qRT-PCR, using SYBR Green Master Mix (Applied Biosystems), and the ABI PRISM 7900 Sequence Detection System (Applied Biosystems Inc, Foster City, CA), as previously described [17]. The primer sequences used in this study are all listed in Table S2.

### Transfection Experiments

The biological effects of HFE were investigated using siRNAs targeting HFE. siHFE1 (Hs_HFE_5 FlexiTube siRNA) and siHFE2 (Hs_HFE_2 FlexiTube siRNA) were purchased from Qiagen. A scrambled siRNA (Hs_Control_ss Flexitube siRNA) served as a negative control. All transfections were performed in complete media without antibiotics using Lipofectamine 2000 and 20 nM siRNA, as previously described [18].

### Reagents

Ciclopirox olamine (CPX), Deferoxamine mesylate salt (DFO), Ferric Ammonium Citrate (FAC) were obtained from Sigma-Aldrich.

### Viability and Clonogenic Assays

The viability of HNSCC cells transfected with siHFE + radiation (RT), or cells treated with CPX, was determined using CellTiter 96 Non-Radioactive Cell Proliferation Assay (MTS) (Promega BioSciences), according to the manufacturer’s recommendations. The colony forming ability of HNSCC cells transfected with siHFE + RT, or cells treated with CPX + RT was determined using the clonogenic assay as previously described [19]. Briefly, 48 hours after transfection with siHFE or 72 hours after treatment with CPX, FaDu cells were re-seeded in 6-well plates, and incubated at 37^0^C under 5% CO_2_ for 10-12 days. The plates were then washed and stained with 0.1% crystal violet in 50% methanol, and the number of colonies was then counted. The fraction of surviving cells was calculated by comparison of siHFE vs. siCTRL or CPX vs. CTRL.

### Flow Cytometry

Flow cytometry analyses were performed on a FACSCalibur Flow Cytometer (BD Biosciences), analyses were performed using FlowJo 7.5 software (Tree Star, San Carlos, CA, USA), as previously described [17].

### Labile Iron

The cellular labile iron pool was measured using calcein-acetoxymethylester (calcein-AM) as specified by the manufacturer (Invitrogen). Transfected cells were incubated with 1 uM of calcein-AM for 15 minutes at 37°C. Cells were washed with PBS, then measured by flow cytometry, as previously described [18].

### BrdU Incorporation

BrdU incorporation was measured using Exalpha Biological BrdU Colorimetric ELISA Kit. Briefly, transfected cells were incubated with the BrdU reagent for 24 hours, fixed, stained and analyzed according to the manufacturer’s specifications, as previously described [18].

### ROS Experiments

Intracellular reactive oxygen species (ROS) levels was measured using the non-specific 5-(and 6-) chloromethyl-2′,7′-dichlorodihydrofluorescein diacetate (CM-H2DCFDA; excitation 488 nm, emission 525 nm) as instructed by the manufacturer (Invitrogen). Transfected cells were incubated with 5 uM of CM-H2DCFDA for 30 minutes at 37°C. Cells were washed with PBS, then measured by flow cytometry [18].

### Western Blot

FaDu cells were transfected with siHFE or control, 48 hours post-transfection, cells were lysed in 1M Tris-HCl (pH 8), 5M NaCl, and 1% NP40 plus the protease inhibitor cocktail (Roche Diagnostics). Protein concentration was assessed as previously described [17]. The membranes were probed with anti-B-Catenin rabbit monoclonal antibody (Cell Signalling, 8814) or anti-HFE monoclonal antibody (Abnova) followed by secondary antibodies conjugated to horseradish peroxidase (Abcam). GAPDH and α-tubulin protein expression were used as loading controls. Western blots were quantified with the Adobe Photoshop Pixel Quantification Plug-In (Richard Rosenman Advertising & Design).

### Iron Rescue Experiments

FaDu cells were transfected with siHFE or control; 24 hours post transfection, cells were treated with either 5µM of DFO, 5µM of FACS or negative control (DMSO). Forty-eight hour post-transfection, FaDu cells were treated with or without 2 Gy of RT. Five days post-transfection, cell viability was measured as described above.

### Tumour Formation Assay

FaDu cells were transfected with siCTRL or siHFE. Forty-eight hours later, viable cells were harvested and 2.5x10^5^ cells were suspended in 100 µL of media, and injected intramuscularly into the left gastrocnemius muscle of 8-10 week old female severe combined immunodeficient (SCID) BALB/c mice. Tumour growth was monitored by measuring the tumor plus leg diameter (TLD) two to three times per week; mice were sacrificed once the TLD reached 13 mm as a humane end-point.

### Tumour Formation Assay with CPX

For the CPX study, two weeks following FaDu tumour cell implantation as described above, mice were treated daily from Monday to Friday by oral gavage with CPX (25 mg/kg) in water or vehicle control for a total of two weeks. Tumour growth was monitored by measuring the tumor plus leg diameter (TLD) three times per week; mice were sacrificed once the TLD reached 13 mm as a humane end-point.

### Immunohistochemistry of Iron Proteins

Expression of TFR1 and HFE was evaluated in 26 primary diagnostic HNSCC biopsy sections using microwave antigen retrieval in combination with the Level-2 Ultra Streptavidin System, and anti-HFE (Sigma HPA017276, 1/300 dilution), or anti-TFR1 (Sigma HPA028598, 1/500 dilution), as previously described [17]. Briefly, 4-um sections were deparaffin, treated with an antigen retrieval reagent, blocked with 3% hydrogen peroxide and incubated with either anti-HFE or anti-TFR1 at 4°C overnight. The following day, sections were incubated with a biotinylated secondary antibody and streptavidin to complete the staining. Cytoplasmic staining of anti-HFE or anti-TFR1 was scored from 0 to 3 based on the staining intensity which was defined accordingly: 0 (no staining); 1 (mild increased staining compare to the corresponding normal epithelium); 2 (moderate increased staining) and 3 (intense increased staining).

### Statistical Analysis

All experiments have been performed at least three independent times, and the data are presented as the mean + standard error of mean (SEM). The comparison between two treatment groups was analyzed using the Student’s *t* test. Two-sided tests were applied. Results were considered significant if the p-value was less than or equal to 0.05. Analysis and graphs were completed using Graphpad Prism Software (Graphpad Software, Inc).

## Results

### HFE is overexpressed in HNSCC cell lines compared to the NOE cell line

**Figure 1:**
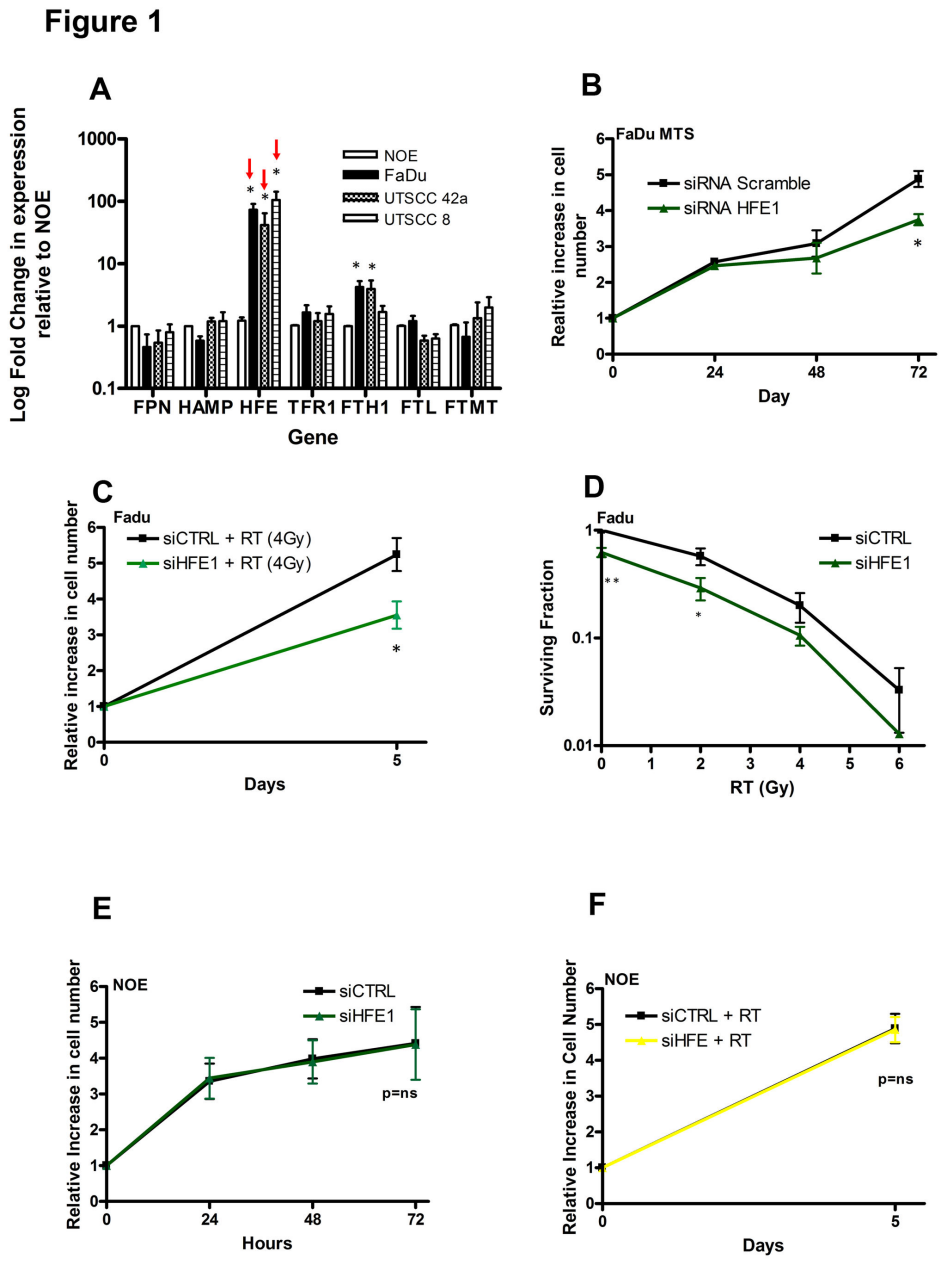
HFE is overexpressed in HNSCC and knockdown preferentially reduced viability and clonogenicity in HNSCC cells compared to NOE cells. (A) qRT-PCR analysis of FPN, HAMP, HFE, TFR1, FTH1, FTL and FTMT expression in FaDu, UTSCC 42a and UTSCC8 HNSCC cancer cell lines, normalized to those genes in NOE cells. (B) FaDu cells were transfected with 20 nM of siCTRL or siHFE1. Cell viability was assessed in FaDu cells by the MTS assay 24-72 hours post-transfection. (C) FaDu cells were transfected with 20 nM of siCTRL or siHFE1, then irradiated 48 hrs post-transfection (4 Gy). Cell viability was assessed by the MTS assay 5 days post-transfection. (D) Clonogenic survival of FaDu cells was measured 10 to 12 days after re-seeding cells treated with siCTRL (20 nM) or siHFE1 (20 nM) for 72 hours. (E) Cell viability of NOE cells was assessed by MTS assay 24, 48 and 72 hrs after transfection with 20 nM of siCTRL or siHFE1. (F) NOE cells were transfected with 20 nM of siCTRL or siHFE1 or 2?, then irradiated 48 hrs post-transfection (4 Gy). Cell viability was assessed by the MTS assay 5 days post transfection. *P<0.05, **P <0.005, P = ns (not significant).

To identify differentially-expressed iron regulating genes in HNSCC, basal mRNA expression levels in three HNSCC cell lines was compared to those in a NOE using qRT-PCR. HFE was noted to have the highest expression level of >80-fold overexpression in HNSCCs vs. the NOE cell line (Figure 1A). Ferritin (FTH1) had the second highest level of overexpression in the FaDu and UTSCC 42a cells, compared to the NOE. Of note, TFR1 also appeared to be slightly overexpressed in HNSCC compared to the NOE cell line.

### In vitro effects of HFE down regulation

In order to assess the biological significance of HFE overexpression, knockdown experiments were performed in HNSCC cells using a siRNA approach. Sustained knockdown was achieved in FaDu cells for up to 72 hours using two independent siRNAs targeting HFE at both the transcript and protein levels (Figures S1A and S1B). HNSCC cells demonstrated a significant reduction in cell viability with or without radiation after transfection with siHFE compared to siCTRL (Figure 1B-C, S1C-E). Furthermore, the ability of HNSCC cells to form colonies was significantly reduced with or without radiation after transfection with siHFE compared to the siCTRL (Figure 1D, S1F-G). In contrast, viability of NOE cells with or without radiation remained unchanged after transfection with siHFE compared to the siCTRL (Figure 1E-F, S1H). Overall, these observations demonstrated that decreasing HFE preferentially reduced viability and clonogenicity in HNSCC compared to NOE cells. To better understand the mechanism(s) responsible for mediating this phenotype, we investigated the ability of HFE to regulate cellular iron.

### HFE regulated HAMP and the labile iron pool in HNSCC cells

**Figure 2:**
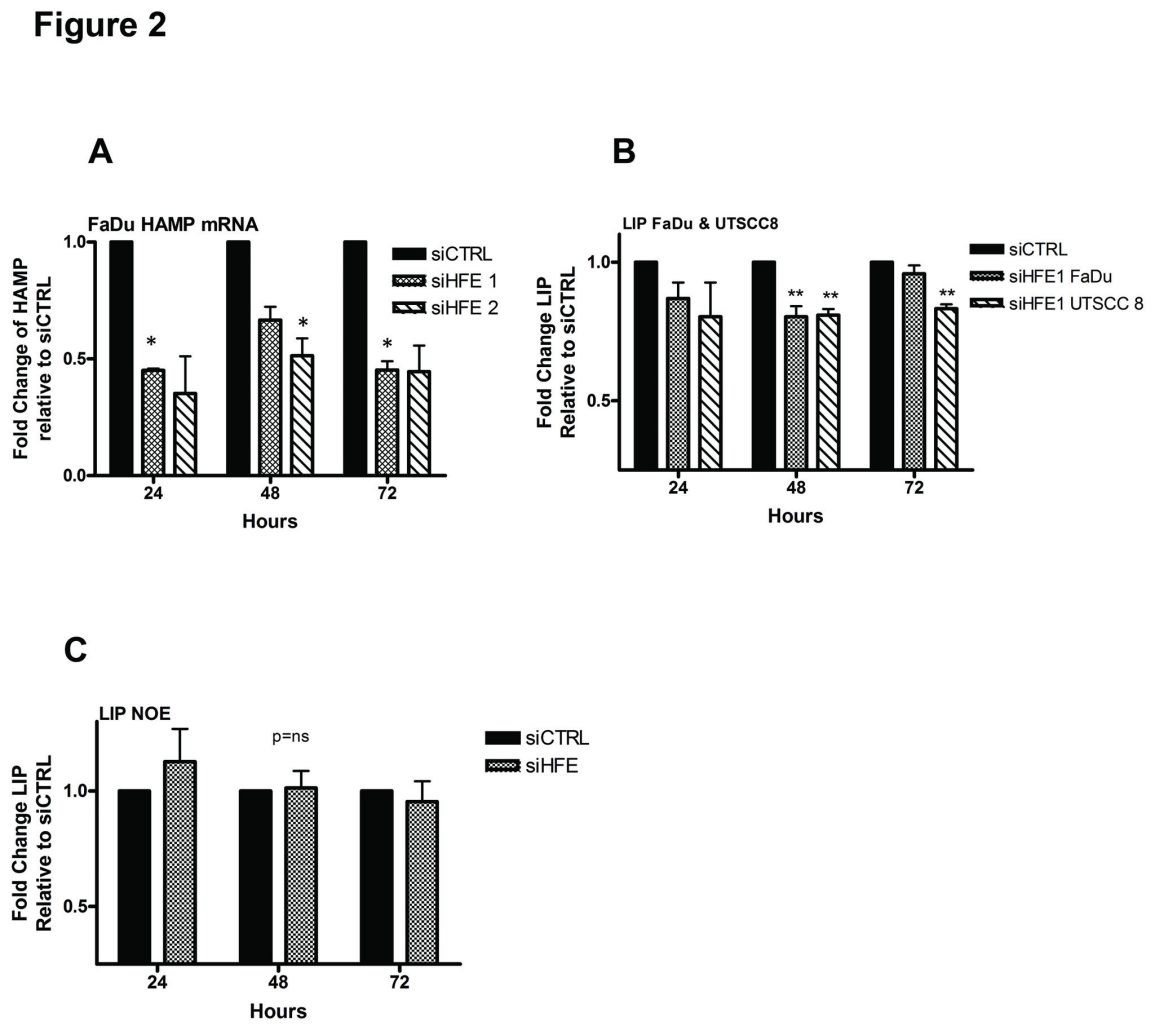
HFE regulates cellular iron. (A) qRT-PCR of HAMP levels at 24, 48 and 72 hrs post-transfection with 20 nM each of siHFE1, or siHFE2, relative to fold-change in siCTRL-treated cells. (B) Cellular labile iron pool (LIP) in FaDu and UTSCC8 cells transfected with 20 nM each of siCTRL or siHFE, detected by flow cytometry with calcein-AM at 24-72 hrs post-transfection. (C) Cellular LIP of NOE cells transfected with 20 nM of siCTRL or siHFE, detected by flow cytometry with calcein-AM at 24-72 hrs post transfection. *P<0.05, **P <0.005, P=ns (not significant).

To determine if HFE was involved in regulating hepcidin (HAMP), we measured mRNA levels of HAMP by qRT-PCR after transfection with siHFE. FaDu cells demonstrated a significant decrease in HAMP mRNA transcript level (<0.5-fold) for up to 72 hours post-transfection with siHFE compared to the siCTRL (Figure 2A). In addition, the labile iron pool (LIP) was also significantly decreased (by ~20%) after HFE knockdown in HNSCC cells compared to siCTRL (Figure 2B). In contrast, there was no significant change in the LIP in NOE cells with siHFE transfection (Figure 2C). Overall, these experiments demonstrated the ability of HFE to alter cellular iron levels preferentially in HNSCC compared to NOE cells. To determine if intracellular iron levels were involved in mediating these siHFE phenotypes, we performed a series of iron rescue experiments.

### Iron mediates the cell proliferation of HFE

**Figure 3:**
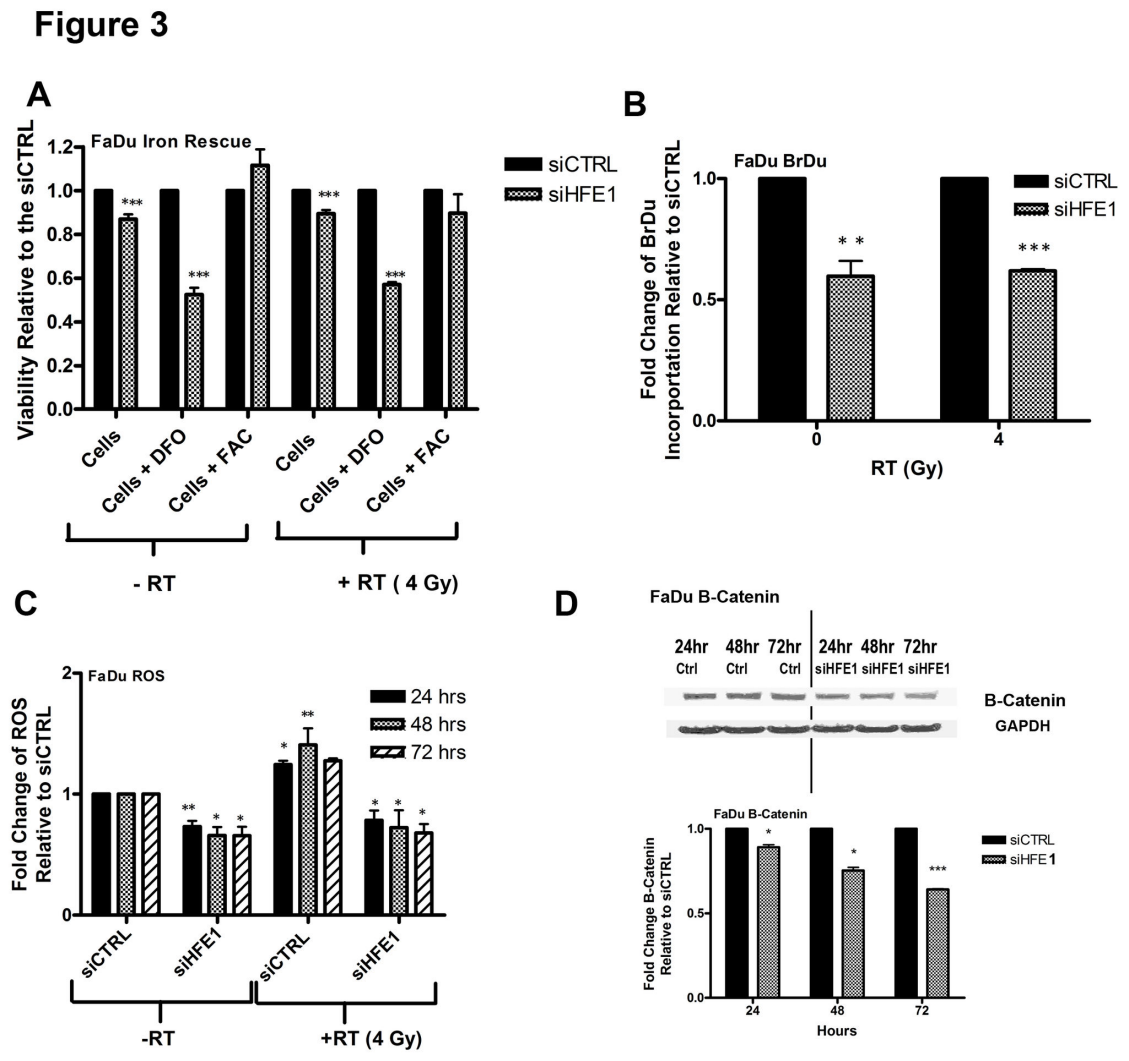
Iron is a critical mediator of the cellular effects of HFE knockdown (A) FaDu cells were transfected with 20 nM each of siCTRL or siHFE1, then treated with 5 uM of DFO, or 5uM of FAC, 24 hrs post-transfection, followed by ± RT (4 Gy), 48 hrs post-transfection. Cell viability was assessed by the MTS assay 5 days post-transfection. (B) BrdU incorporation was assessed in FaDu cells 5 days after transfection with 20 nM each of siCTRL or siHFE1, ± RT (4 Gy, 48 hrs post-transfection). (C) FaDu cells were transfected with 20 nM each of siCTRL or siHFE, ± RT (4 Gy, 48 hrs post-transfection). Total cellular ROS level was detected by flow cytometry with CM-H2DCFDA 24-72 hrs post-RT. (D) Western blotting of B-catenin was measured in FaDu cells 24-72hr post transfection with siCTRL (20 nM) or siHFE1 (20 nM); images (above), quantification (below). *P<0.05, **P<0.005, ***P<0.0005, P=ns (not significant)

Cell viability was measured in HNSCC cells treated with siHFE alone, siHFE with an iron chelator deferoxamine (DFO), or siHFE combined with soluble iron (FAC), both with and without RT (Figures 3A & S2A-B). These studies demonstrated that the addition of DFO further reduced viability of HNSCC cells after HFE knock-down, which was completely rescued by the addition of FAC; RT had no significant differential effect on this process. These findings confirmed that iron was a critical mediator of these effects. We then proceeded to evaluate the effects of siHFE on downstream iron-dependent processes.

The BrdU incorporation assay was employed to measure changes in DNA synthesis. HNSCC cells transfected with siHFE demonstrated a significant reduction (60%) in BrdU incorporation compared to siCTRL-treated cells (Figure 3B, S2C). In contrast, no significant change in BrdU incorporation was observed for NOE cells transfected with siHFE compared to siCTRL-treatment (Figure S2D). Flow cytometry was used to measure changes in ROS levels after siHFE. As expected, FaDu cells demonstrated a significant reduction in ROS levels after transfection with siHFE compared to siCTRL, both with and without RT (Figures 3C and S2E). Lastly, the Wnt pathway was examined by analyzing changes in B-Catenin. FaDu cells transfected with siHFE demonstrated a significant reduction (~40%) in B-Catenin levels for up to 72 hours compared siCTRL-treated cells (Figure 3D).

### siHFE marginally reduces tumour formation in vivo

Next, we assessed the effects of HFE knock-down in an *in vivo* tumour model. Tumorigenicity was measured *in vivo* using SCID mice injected intra-muscularly with FaDu cells transfected with siHFE or siCTRL. Suppression of siHFE marginally decreased tumour formation compared to the negative control, noticeable at later time points (>27days) (Figure S2F).

### Iron chelator CPX reduced cell proliferation in HNSCC cell lines

**Figure 4:**
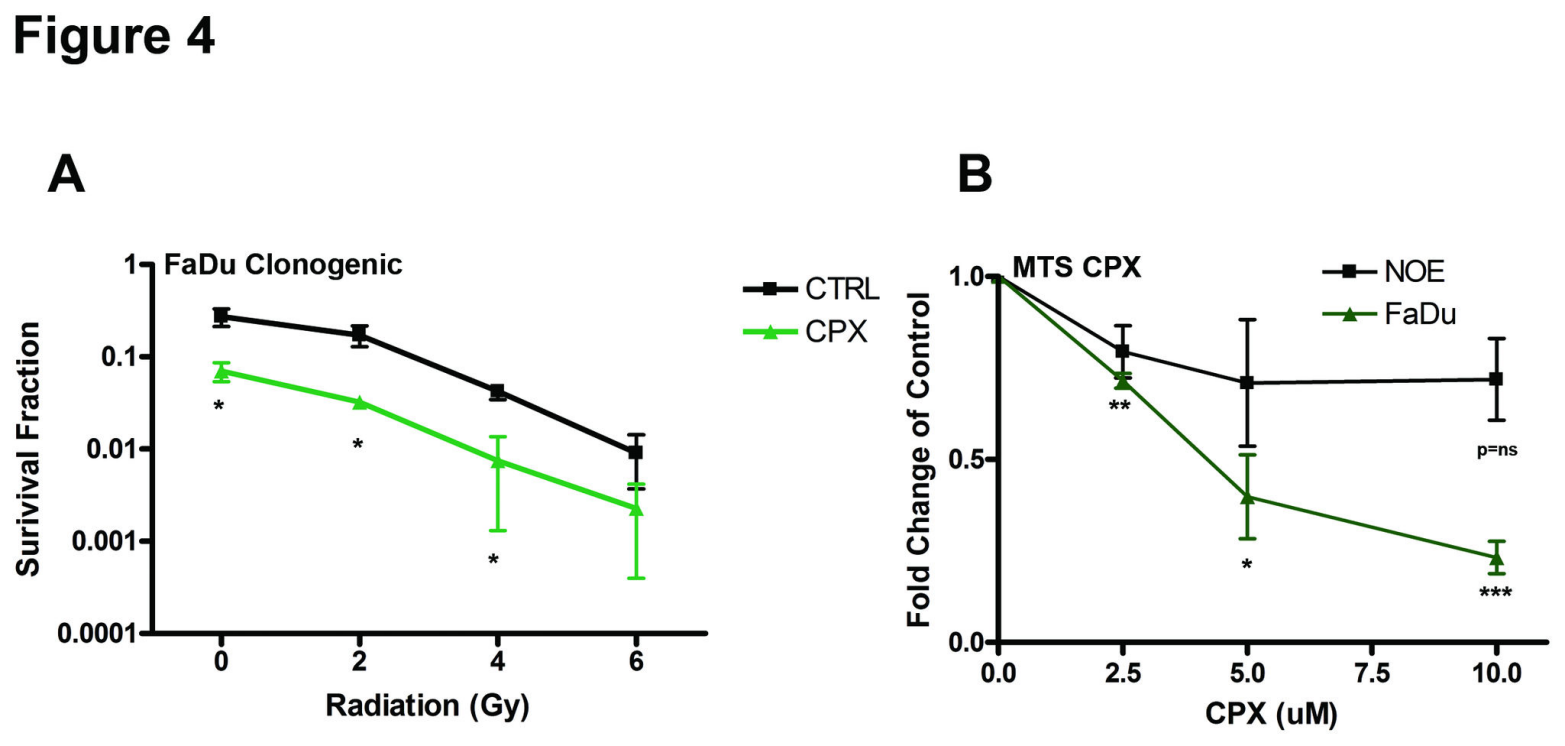
Ciclopirox olamine reduced HNSCC cell viability and clonogenicity. (A) Clonogenic survival of FaDu cells was measured 10 to 12 days after re-seeding of cells that were treated with ethanol (5 uM) or CPX (5 uM) for 72 hours, followed by RT (0, 2, 4 or 6 Gy). (B) Cell viability of FaDu and NOE cells was assessed by MTS assay 72 hrs after treatment with CPX (2.5 uM, 5 uM or 10 uM). *P<0.05, **P<0.005, ***P<0.0005, P=ns (not significant).

Given the challenges in the therapeutic application of a siRNA strategy, HNSCC cells were treated with a clinically-approved iron chelator, Ciclopirox olamine (CPX), to determine if the siHFE phenotype could be recapitulated. Treatment of HNSCC cell lines with 5 uM of CPX resulted in a significant reduction in colony formation, with or without RT, compared to vehicle-treated cells (Fig 4A, S3A-B). In contrast, CPX had a much lesser effect on the viability of NOE compared to FaDu cells (Figure 4B). Unfortunately, the administration of CPX for 2 weeks at 25 mg/kg failed to reduce tumour growth in the FaDu xenograft model (Figure S3C).

### Iron proteins are de-regulated in primary HNSCC tissue samples

**Figure 5:**
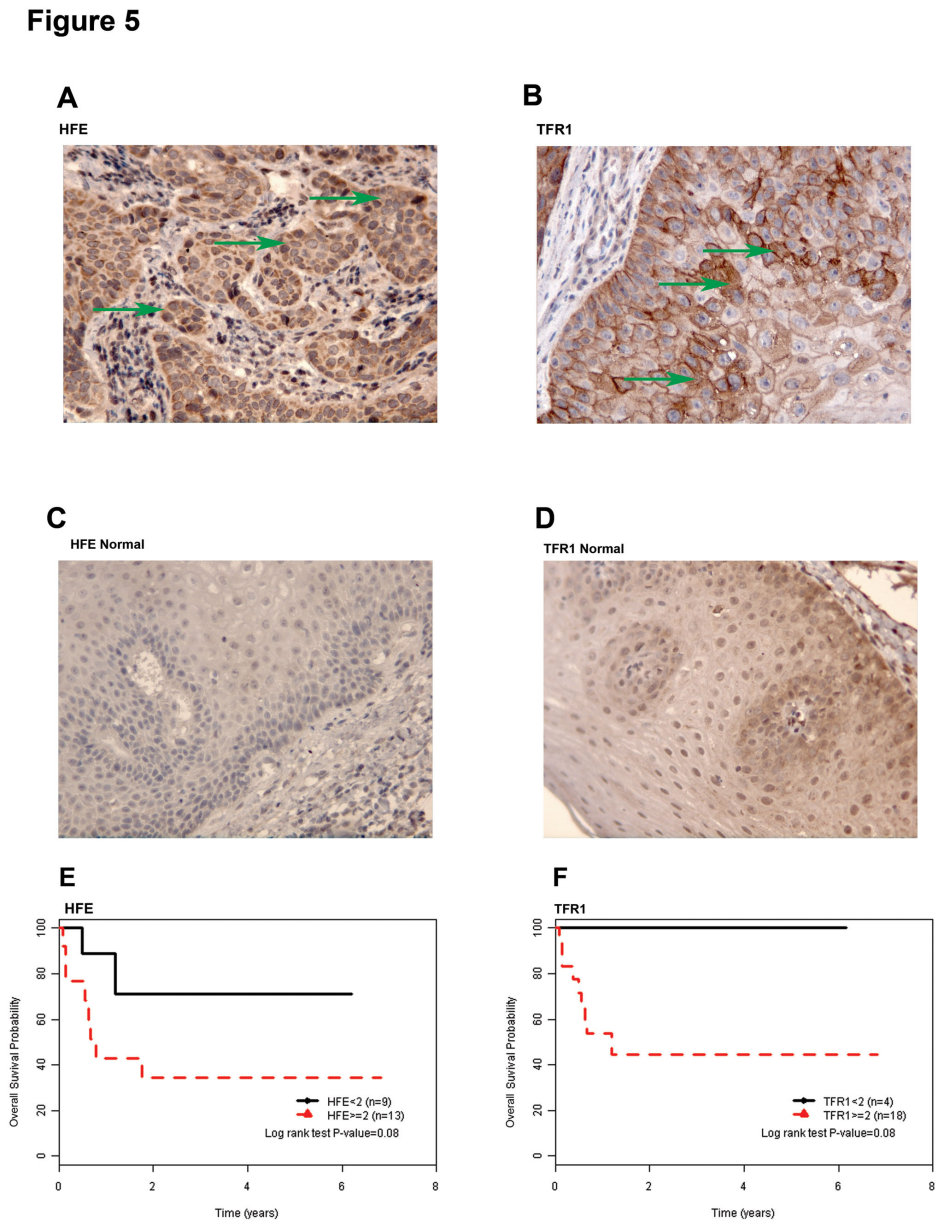
HFE and TFR1 expression in HNSCC patient samples. (A) A representative image of HFE immunohistochemical expression from a primary HNSCC biopsy; arrows indicating tumor cells exhibiting cytoplasmic signal. (B) A representative image of TFR1 immunohistochemical expression from a primary HNSCC biopsy; arrows indicating tumor cells exhibiting cytoplasmic membrane signal. (C) A representative image of HFE immunohistochemical expression from a normal larynx. (D) A representative image of TFR1 immunohistochemical expression from a normal larynx. (E) Kaplan-Meier plot of overall survival for HNSCC patients dichotomized based on high (≥2) *vs*. low (<2) HFE immunohistochemistry scores. (F) Kaplan-Meier plot of overall survival for HNSCC patients dichotomized based on high (≥2) *vs*. low (<2) TFR1 immunohistochemistry scores.

Immunohistochemistry (IHC) was utilized to visually confirm the expression of HFE and TFR1 in HNSCC tissues. Intense immuno-expression of both HFE and TFR1 was observed in the cytoplasm of tumour cells, but not in the adjacent stroma or infiltrating lymphocytes (Figure 5A and 5B). In contrast, minimal immuno-expression of HFE and TFR1 was observed in a normal larynx (Figure 5C-D), confirming the higher expression of HFE and TFR1 in HNSCC vs. normal tissues. When the expression of HFE or TFR1 were dichotomized between high (IHC ≥2) *vs.* low (IHC <2) levels, the former groups experienced a shorter overall survival compared to the latter (Figure 5E-F; p=0.08); although statistical significance was not attained due to the small sample size. Similarly, the median IHC score for both HFE and TFR1 was higher in the relapsed vs. non-relapsed patient samples (Figure S4A-B), but was statistically significant only for HFE expression (p=0.04).

## Discussion

**Figure 6:**
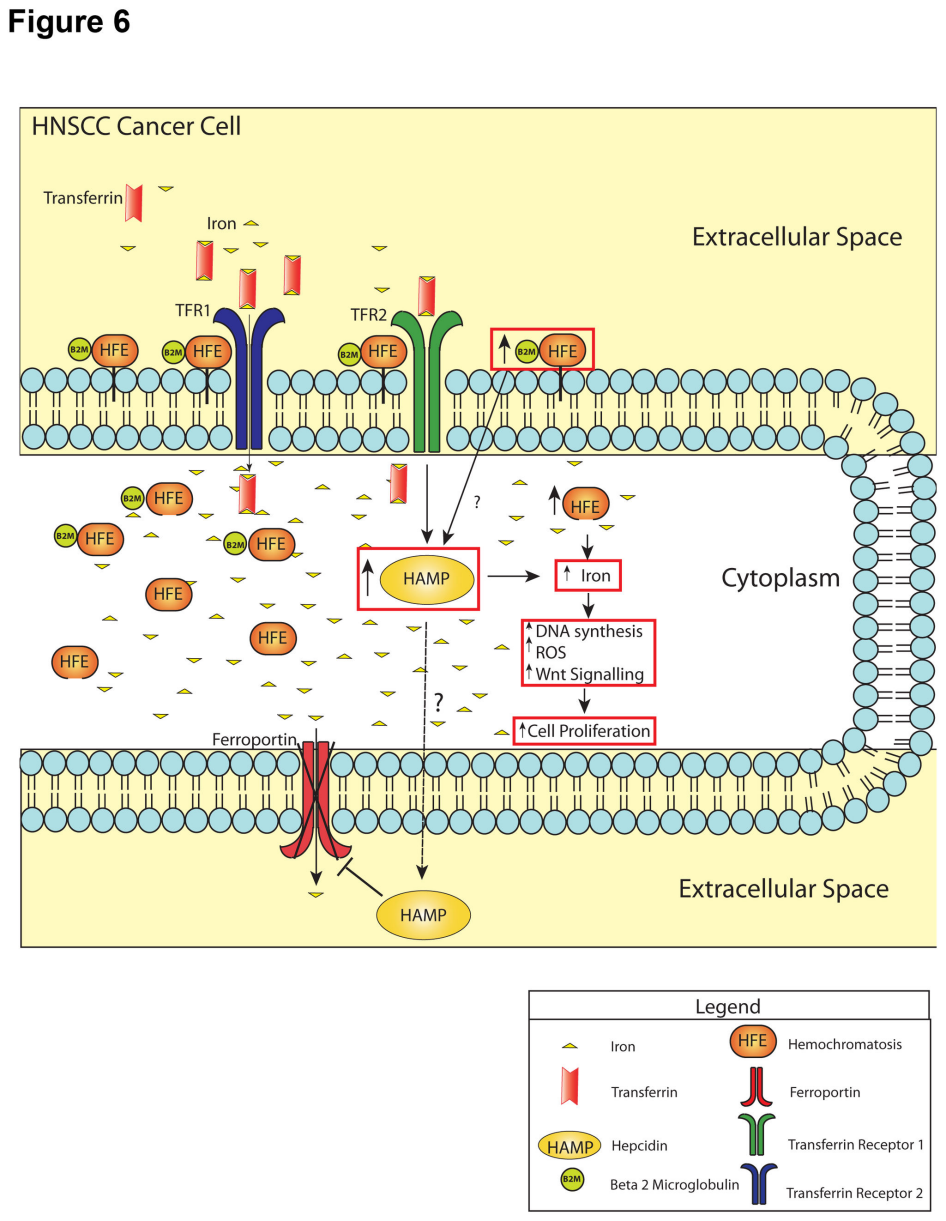
Proposed model for the role HFE in promoting HNSCC progression *via* iron regulation. Schema showing that HFE overexpresion leads B2M binding for cell membrane trafficking. HFE increases HAMP either directly or via TFR2. HAMP then exits the cell, through an unknown mechanism, and in turn degrades FPN, which leads to iron accumulation. Collectively, this results in increased DNA synthesis, elevated ROS, and Wnt signaling, all driving tumour cell proliferation and clonogenicity. Boxes in red denote the data demonstrated in this current study.

Herein, we report for the first time that HFE overexpression appears to be a novel mechanism responsible for HNSCC disease progression. Overexpression of HFE in HNSCC leads to increased hepcidin, which in turn promotes intracellular iron accumulation, resulting in increased DNA synthesis, elevated ROS levels, Wnt signalling, all driving tumour cell proliferation and clonogenicity, with iron as a critical mediator in this process (Figure 6). One potential therapeutic strategy in this context is utilization of an iron chelator ciclopirox olamine (CPX), which preferentially reduced clonogenic survival and viability in HNSCC cells, with minimal effects on the NOEs. Moreover, HFE and TFR1 overexpression demonstrated a trend towards worse outcome, which collectively document the critical role of iron deregulation in HNSCC progression.

Additional evidence of the relevance of these iron regulating genes are provided by examination of publicly-available HNSCC databases (www.oncomine.org) [20], confirming significant overexpression of both HFE [21], and TFR1 [21–23] in HNSCC patient samples, demonstrating that this is indeed a commonly dysregulated pathway in this disease. Moreover, HFE was also overexpressed in other cancers including brain [24], and renal cell carcinomas [25]. To identify potential mechanism(s) leading to their overexpression, the TCGA HNSCC database using the cBIO Cancer Genomic Portal software [26] was interrogated by comparing tumour transcript levels to DNA copy number in 295 discrete patient datasets. The majority of these HNSCCs were diploid for *HFE*; hence chromosomal alteration did not appear to be responsible for its overexpression. However, amplification of the *TFR1* gene was observed in 18% of HNSCC samples, which corresponded to elevated TFR1 mRNA expression levels, indicating genomic alteration as one mechanism for TFR1 overexpression in HNSCC. Given the complex network of proteins involved in iron regulation [27], it is clear that multiple mechanisms are responsible for iron deregulation in human cancers. For instance mTOR, which is frequently activated in HNSCC [28] has been recently linked to TFR1 stability and iron regulation [29], providing yet another mechanism for iron deregulation in HNSCC. Hence, there are likely several different mechanisms accounting for HFE overexpression in HNSCC, resulting in iron perturbation.

Hemochromatosis (HFE) is a transmembrane glycoprotein, broadly expressed throughout the human body [30]; one of its principal roles is to regulate hepcidin (HAMP) [8], which in turn, internalizes and degraded ferroportin (FPN) (see Figure 6) [10]. HAMP somehow exits the cell, then binds to FPN at the plasma membrane, causing tyrosine phosphorylation leading to the internalization of FPN. Once internalized, FPN is de-phosphorylated, then ubiquitylated and degraded through the lysosomal pathway [31]. Ultimately, degradation of FPN by HAMP leads to intracellular retention of iron.

Under physiological conditions, HAMP is presumably secreted by the liver in response to changes in plasma iron levels. However, recent evidence suggests that HAMP may play a pathological role in human malignancies; for example, low FPN and high HAMP have been associated with poor prognosis in breast cancer [32]. Elevated HAMP mRNA levels correlated with low FPN expression in colorectal carcinoma [33]. The precise mechanism(s) whereby elevated HAMP contributes to carcinogenesis remains to be elucidated; however it is conceivable that HAMP could be secreted by cancer cells to degrade FPN, thereby increasing intracellular iron levels, as suggested by our data. In fact, elevated serum HAMP levels have been associated with renal cell carcinoma metastases [34]; as well, high urinary HAMP levels have been observed in multiple myeloma patients [35], both suggesting pathologic secretion of HAMP by the cancer cells. Furthermore, HeLa cells transiently transfected with a plasmid containing FPN and exposed to HAMP, resulted in internalization of FPN [10], demonstrating that tumours respond to HAMP in a similar manner as hepatocytes or macrophages.

The iron regulatory network contains over 151 chemical species and 107 reactions steps [27], thus is tightly regulated. The phenomenon of cancer cells requiring more iron to maintain their high cellular turnover and DNA synthesis is observed in many different malignancies. As a result, the iron network is often deregulated in cancers to accommodate for this increased iron demand. TFR1 overexpression has been described in a wide range of tumour vs. normal tissues, including breast [36], esophageal [37] and lung [38], providing one mechanism to import more iron into the cell. Overexpression of ferritin, the central storage molecule of iron has also been reported in breast [39], prostate [40], and colon cancer [41], again, underscoring the multiple mechanisms by which cancer cells can accumulate iron to great abundance.

In a study of breast cancer, a more favourable outcome was described in patients with low TFR1 and high HFE gene expression [42]. The authors reasoned that this combination could prevent cellular iron absorption, although this was not experimentally validated. HFE bound to TFR1 can certainly compete with Fe-TF, thereby decreasing iron absorption into cells [7]; however, this might be a cell line specific phenomenon, since HFE overexpression actually stabilizes iron absorption in macrophages [43]. In our current study, HFE upregulated the expression of HAMP (Figure 2A), leading to iron accumulation (Figure 2B), and inhibition of iron release [11,12]. Therefore, although low TFR1 with high HFE could contribute to a net reduction in iron absorption, this effect is likely counterbalanced by the ability of HFE to stimulate HAMP, thereby inhibiting iron release from the cell [11,12].

Since iron is required for a number of cellular processes that are involved in tumour biology such as ROS generation, Wnt signalling and DNA synthesis [4], we reasoned that HFE knockdown would affect all these pathways, which was indeed the case, wherein siHFE significantly reduced ROS level (Figure 3C, S2E). Through Fenton chemistry, ROS are generated when H_2_O_2_ reacts with Fe^2+^ to produce Fe^+3^ and a hydroxyl radical [44]; hence lower iron levels would lead to decreased ROS production. Conversely, elevated ROS levels resulted in increased cell proliferation, and genetic instability [45], both hallmarks of human cancer. A previous study observed that sustained ROS levels promoted progression of HNSCC cell lines [46]; hence, elevated HFE will lead to increased ROS levels, thereby contributing to HNSCC cell aggressiveness.

Recent evidence has also uncovered a role for iron in Wnt signalling [4], corroborated in our current study whereby B-Catenin was suppressed following HFE knock down (Figure 3D). In HNSCC, Wnt overexpression has been described to contribute to HNSCC disease progression [47]. Hence, Wnt is yet another downstream pathway by which elevated HFE can lead to proliferation in HNSCC.

Evaluation of siHFE in an *in vivo* setting was unfortunately not efficacious (Figure S2F); likely due to the limitations of our transient transfection system, and the ability of tumour cells to adapt to HFE knockdown *via* the extensive iron regulatory protein network [27] over the prolonged period of our experiment (> 40 days). Therefore a stable transfection system would have been a better design for this experiment.

In an effort to translate our observation to a potentially feasible therapeutic approach, we sought to exploit a more potent and prolonged system using an existing iron chelator Ciclopirox olamine (CPX) for HNSCC. CPX is already a clinically approved anti-cancer agent, currently being evaluated for AML patients [48]. CPX has been demonstrated to reduce DNA synthesis, cell proliferation [48], and Wnt signalling [4], which have all been demonstrated for our siHFE phenotype. Our data indeed corroborated that HNSCC cells treated with CPX demonstrated a significant reduction in viability, with minimal effects on NOEs (Figure 4B), suggesting cancer specific activity of CPX, which has been reported by other groups [48]. In addition, CPX appeared to achieve a slight degree of radiosensitization, particularly at the higher doses (6 Gy), which is encouraging given the important curative role of radiation therapy in HNSCC.

The observed radiosensitization is an important consideration since ionizing radiation achieves its therapeutic effect largely through the production of hydroxyl radicals and ROS, causing DNA double-strand breaks, along with apoptosis. Iron chelators such as DFO decrease ROS and have been shown to hinder the efficacy of RT [49]. Fortunately, no radio-protective effects were observed with either siHFE or CPX in the HNSCC models (Figures 1C-D and S1E and S3A-B). One could speculate that the reduction in proliferation exceeded the ROS inhibitory effect of the combined treatments. Moreover, DFO directly targets iron whereas siHFE and CPX indirectly target iron, through HAMP and ribonucleotide reductase, thereby avoiding the potential radio-protective properties of iron chelators.

To evaluate the in vivo efficacy of CPX in HNSCC, we adopted the treatment regimen previously reported for a leukemia model [48]. This treatment regime was unfortunately not efficacious in our HNSCC model (Figure S3C), which could be related to pharmacokinetics, since optimization of the drug concentration and dosing regimens were not undertaken in our solid tumour xenograft model. Furthermore, alternative agents might be better suited for solid tumours; a recent study using oral deferasirox achieved efficacy in lung tumour xenografts [50]. However, radioprotection would need to be ruled out before deferasirox could be evaluated in combination with ionizing radiation for HNSCC.

Finally, higher levels of both HFE and TFR1 were observed in primary HNSCC patient samples (Figure 5A-B), with a trend towards worse outcome (Figure 5E-F) and a higher risk of relapse (Figure S4A-B) for patients with higher HFE and TFR1 levels. Given the function of HFE in regulating HAMP [8], and the central role of TFR1 in transporting iron into cells [7], it would be indeed anticipated that this combination would increase iron availability for cells, thereby leading to treatment resistance.

In summary, we have identified a potentially novel prognostic and oncogenic role for HFE in HNSCC, whereby elevated HFE increases HAMP, in turn elevating intracellular iron, leading to cell proliferation and tumour formation through activation of DNA synthesis, Wnt signalling and ROS production (Figure 6). Iron is a critical mediator of this process; hence targeting this network through sustained HFE knock down, or iron chelation strategies definitely warrants further examination as therapeutic avenues for HNSCCs, ensuring maintenance of radio-sensitization.

## Supporting Information

Figure S1 **HFE knockdown reduced cell viability and clonogenicity in FaDu cells with no effect on NOEs.**
(A) qRT-PCR of HFE mRNA expression in FaDu cells 24-72 hours after transfection with siCTRL (20 nM), siHFE1 (20 nM), or siHFE2 (20 nM). (B) Western blotting of HFE was assessed in FaDu cells 24 to 72 hrs post-transfection with siCTRL (20 nM) or siHFE1 (20 nM); images (above), quantification (below). (C) FaDu cells were transfected with 20 nM of siCTRL or siHFE2; cell viability was assessed using the MTS assay 1-3 days post-transfection. (D) UTSCC8 and 42a cells were transfected with 20 nM each of siCTRL or siHFE2; cell viability was assessed by the MTS assay five days post-transfection. (E) FaDu cells were transfected with 20 nM each of siCTRL or siHFE2, and irradiated 48 hrs post-transfection (4 Gy). Cell viability was assessed by the MTS assay 5 days post-transfection. (F) Clonogenic survival of FaDu cells was measured 10 to 12 days after re-seeding of cells treated with siCTRL (20 nM) or siHFE2 (20 nM), then 72 hours later, treated with RT (0, 2, 4, or 6 Gy). (G) Clonogenic survival of UTSCC8 cells was measured 10 to 12 days after re-seeding of cells treated with siCTRL (20 nM) or siHFE1 (20 nM) for 72 hours. (H) Cell proliferation of NOE cells was assessed by MTS assay 1-3 days after transfection with 20 nM each of siCTRL or siHFE2 (black or green, respectively). In addition, cell viability was measure in NOE cells transfected with 20 nM each of siCTRL or siHFE2, followed by RT 48 hrs post-transfection (4 Gy) (yellow and red, respectively). *P<0.05, **P<0.005, ***P<0.0005, P=ns (not significant).
(TIF)

Figure S2 **Iron is a critical mediator of the siHFE phenotype.**
(A) UTSCC8 and UTSCC42a cells were transfected with 20 nM each of siCTRL or siHFE1, then treated with DFO (5 uM) or FAC (5 uM), 24 hrs post-transfection. Cell viability was assessed by the MTS assay 5 days after transfection. (B) FaDu cells were transfected with 20 nM each of siCTRL or siHFE2, then treated with 5 uM each of DFO or FAC, 24 hrs post-transfection, followed by RT (4 Gy) 48 hrs post-transfection. Cell viability was assessed by MTS assay 5 days after transfection. (C) BrdU incorporation was assessed in UTSCC8 cells 5 days after transfection with 20 nM each of siCTRL or siHFE1, + RT (4 Gy, 48 hrs post-transfection). (D) BrdU incorporation was assessed in NOE cells 5 days after transfection with 20 nM each of siCTRL, siHFE1, or siHFE2, + RT (4 Gy, 48 hrs post-transfection). (E) Total cellular ROS level was detected by flow cytometry with CM-H _2_DCFDA in FaDu cells transfected with 20 nM each of siCTRL or siHFE2, + RT (4 Gy, 48 hrs post-transfection), assayed at 24, 48, and 72 hrs post-RT. All data points represent the mean value + SEM after three independent experiments. (F) FaDu cell were transfected with siCTRL (20 nM) or siHFE (20 nM), then implanted intramuscularly (IM) 48 hrs later; each group comprised of 12 mice. Tumor plus leg diameter was measured two to three times a week (y-axis). *P<0.05, **P<0.005, ***P<0.0005, P=ns (not significant).
(TIF)

Figure S3 **Ciclopirox olamine reduced HNSCC clonogenicity.**
(A) Clonogenic survival of UTSCC8 cells was measured 10 to 12 days after re-seeding of cells treated with ethanol (5 uM) or CPX (5 uM) and RT (0, 2, 4 or 6 Gy) 72 hours after CPX treatment. (B) Clonogenic survival of UTSCC42a cells was measured 10 to 12 days after re-seeding cells treated with ethanol (5 uM) or CPX (5 uM) and RT (0, 2, 4 or 6 Gy) 72 hours after CPX treatment. (C) FaDu tumors were established in SCID mice; once TLD reached ~8 mm, mice were randomly assigned to vehicle (water) or CPX, administered as oral dosages (25 mg/kg) five times per week for a total of 2 weeks. Each treatment group comprised of at least four mice. *P<0.05 P=ns (not significant).
(TIF)

Figure S4 **HFE and TFR1 IHC immune-expression score between non-relapsed vs. relapsed HNSCC patient samples**.
(A) HFE immunohistochemistry score (1–3), for non-relapsed (n=12) vs. relapsed (n=10) HNSCC patient samples. (B) TFR1 immunohistochemistry score (1–3) for non-relapsed (n=12) vs. relapsed (n=9) HNSCC patient samples.
(TIF)

Table S1 **Clinical details of the 26 HNSCC patients.**
(TIF)

Table S2 **qRT-PCR primer design sequences.**
(TIF)
