# Supplementary figures and images for: Hemochromatosis Enhances Tumor Progression via Upregulation of Intracellular Iron in Head and Neck Cancer
Source: PLoS One. 2013 Aug 26;8(8):e74075. doi: 10.1371/journal.pone.0074075 (PMC3753261; doi:10.1371/journal.pone.0074075)

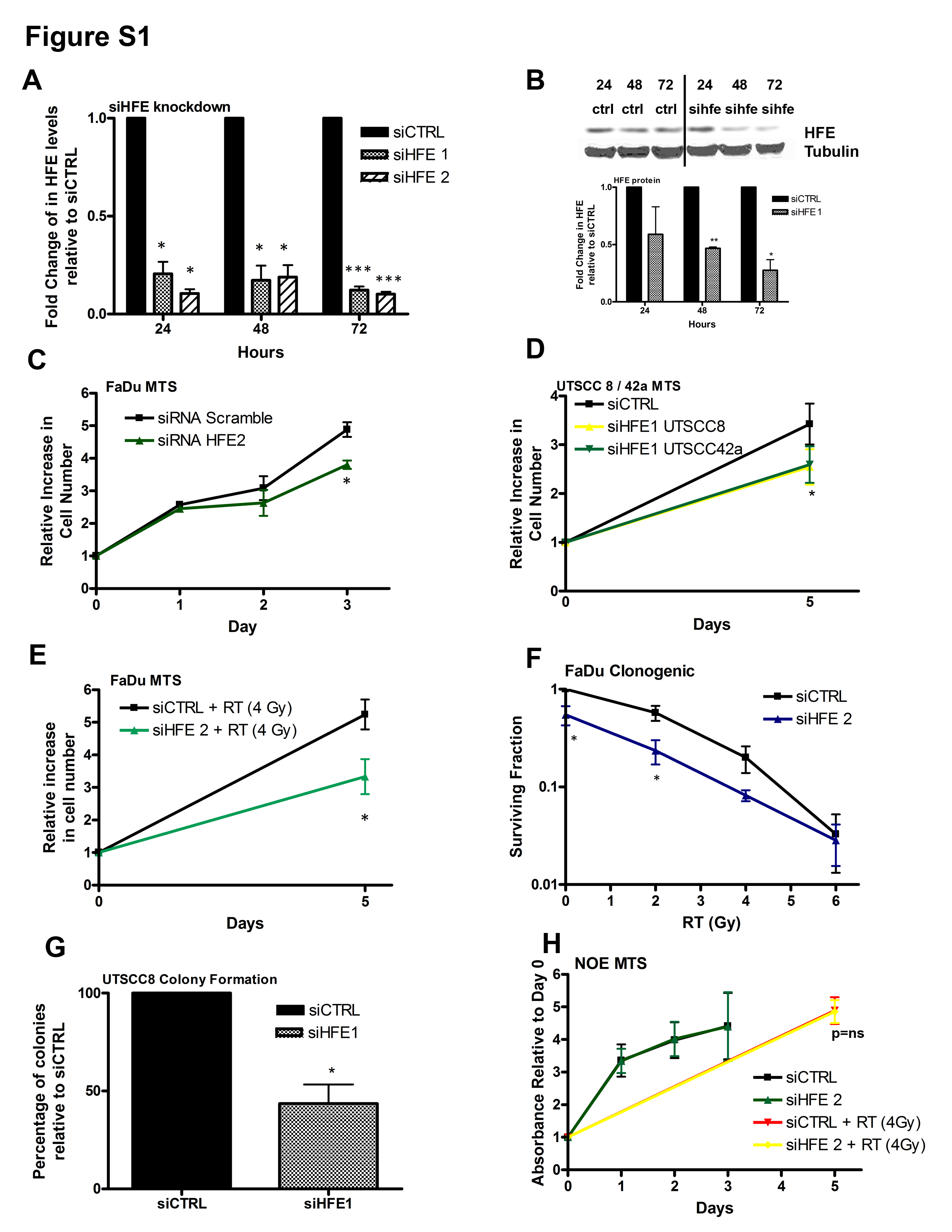

Supplement: Figure S1 — HFE knockdown reduced cell viability and clonogenicity in FaDu cells with no effect on NOEs. (A) qRT-PCR of HFE mRNA expression in FaDu cells 24-72 hours after transfection with siCTRL (20 nM), siHFE1 (20 nM), or siHFE2 (20 nM). (B) Western blotting of HFE was assessed in FaDu cells 24 to 72 hrs post-transfection with siCTRL (20 nM) or siHFE1 (20 nM); images (above), quantification (below). (C) FaDu cells were transfected with 20 nM of siCTRL or siHFE2; cell viability was assessed using the MTS assay 1-3 days post-transfection. (D) UTSCC8 and 42a cells were transfected with 20 nM each of siCTRL or siHFE2; cell viability was assessed by the MTS assay five days post-transfection. (E) FaDu cells were transfected with 20 nM each of siCTRL or siHFE2, and irradiated 48 hrs post-transfection (4 Gy). Cell viability was assessed by the MTS assay 5 days post-transfection. (F) Clonogenic survival of FaDu cells was measured 10 to 12 days after re-seeding of cells treated with siCTRL (20 nM) or siHFE2 (20 nM), then 72 hours later, treated with RT (0, 2, 4, or 6 Gy). (G) Clonogenic survival of UTSCC8 cells was measured 10 to 12 days after re-seeding of cells treated with siCTRL (20 nM) or siHFE1 (20 nM) for 72 hours. (H) Cell proliferation of NOE cells was assessed by MTS assay 1-3 days after transfection with 20 nM each of siCTRL or siHFE2 (black or green, respectively). In addition, cell viability was measure in NOE cells transfected with 20 nM each of siCTRL or siHFE2, followed by RT 48 hrs post-transfection (4 Gy) (yellow and red, respectively). *P<0.05, **P<0.005, ***P<0.0005, P=ns (not significant). (TIF) [file pone.0074075.s001.tif]

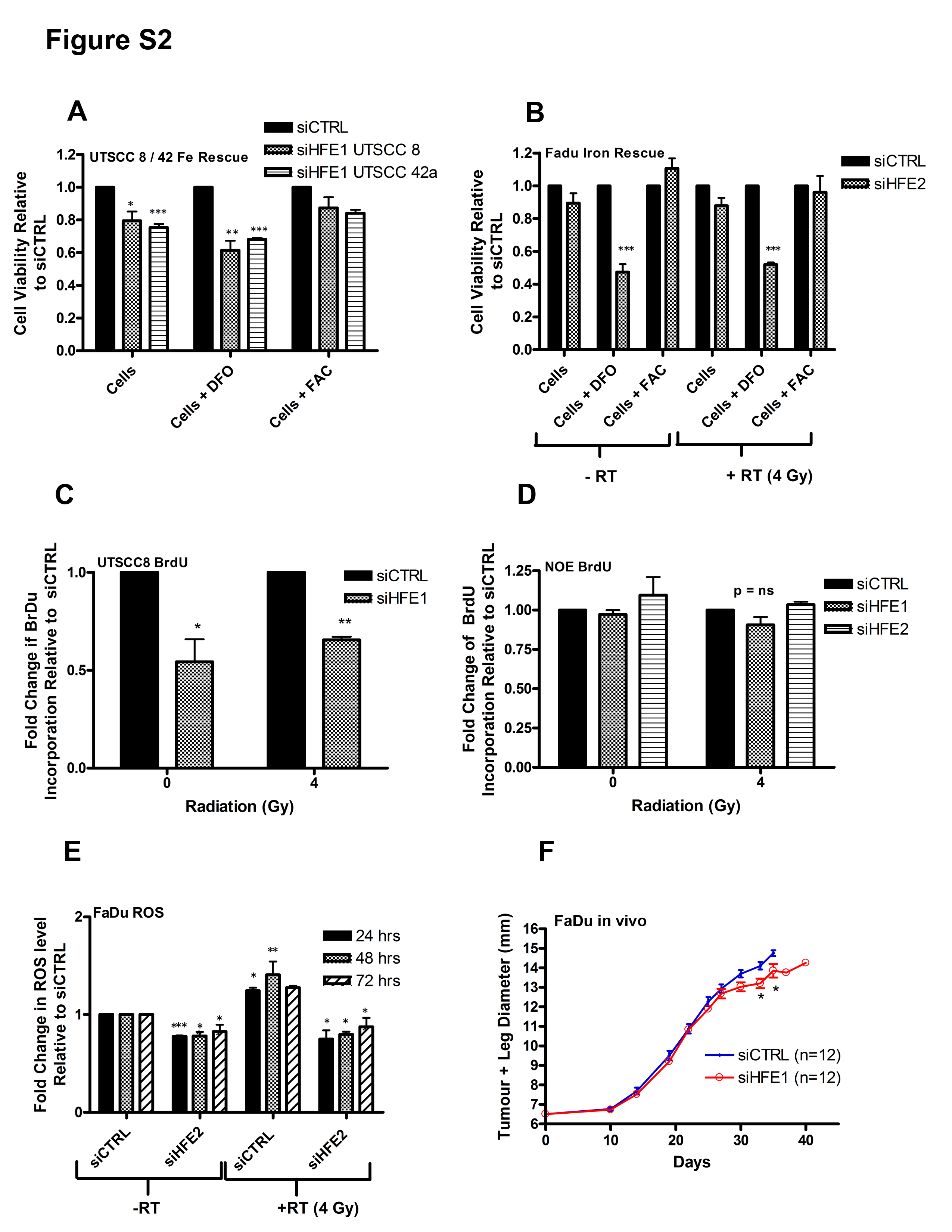

Supplement: Figure S2 — Iron is a critical mediator of the siHFE phenotype. (A) UTSCC8 and UTSCC42a cells were transfected with 20 nM each of siCTRL or siHFE1, then treated with DFO (5 uM) or FAC (5 uM), 24 hrs post-transfection. Cell viability was assessed by the MTS assay 5 days after transfection. (B) FaDu cells were transfected with 20 nM each of siCTRL or siHFE2, then treated with 5 uM each of DFO or FAC, 24 hrs post-transfection, followed by RT (4 Gy) 48 hrs post-transfection. Cell viability was assessed by MTS assay 5 days after transfection. (C) BrdU incorporation was assessed in UTSCC8 cells 5 days after transfection with 20 nM each of siCTRL or siHFE1, + RT (4 Gy, 48 hrs post-transfection). (D) BrdU incorporation was assessed in NOE cells 5 days after transfection with 20 nM each of siCTRL, siHFE1, or siHFE2, + RT (4 Gy, 48 hrs post-transfection). (E) Total cellular ROS level was detected by flow cytometry with CM-H 2DCFDA in FaDu cells transfected with 20 nM each of siCTRL or siHFE2, + RT (4 Gy, 48 hrs post-transfection), assayed at 24, 48, and 72 hrs post-RT. All data points represent the mean value + SEM after three independent experiments. (F) FaDu cell were transfected with siCTRL (20 nM) or siHFE (20 nM), then implanted intramuscularly (IM) 48 hrs later; each group comprised of 12 mice. Tumor plus leg diameter was measured two to three times a week (y-axis). *P<0.05, **P<0.005, ***P<0.0005, P=ns (not significant). (TIF) [file pone.0074075.s002.tif]

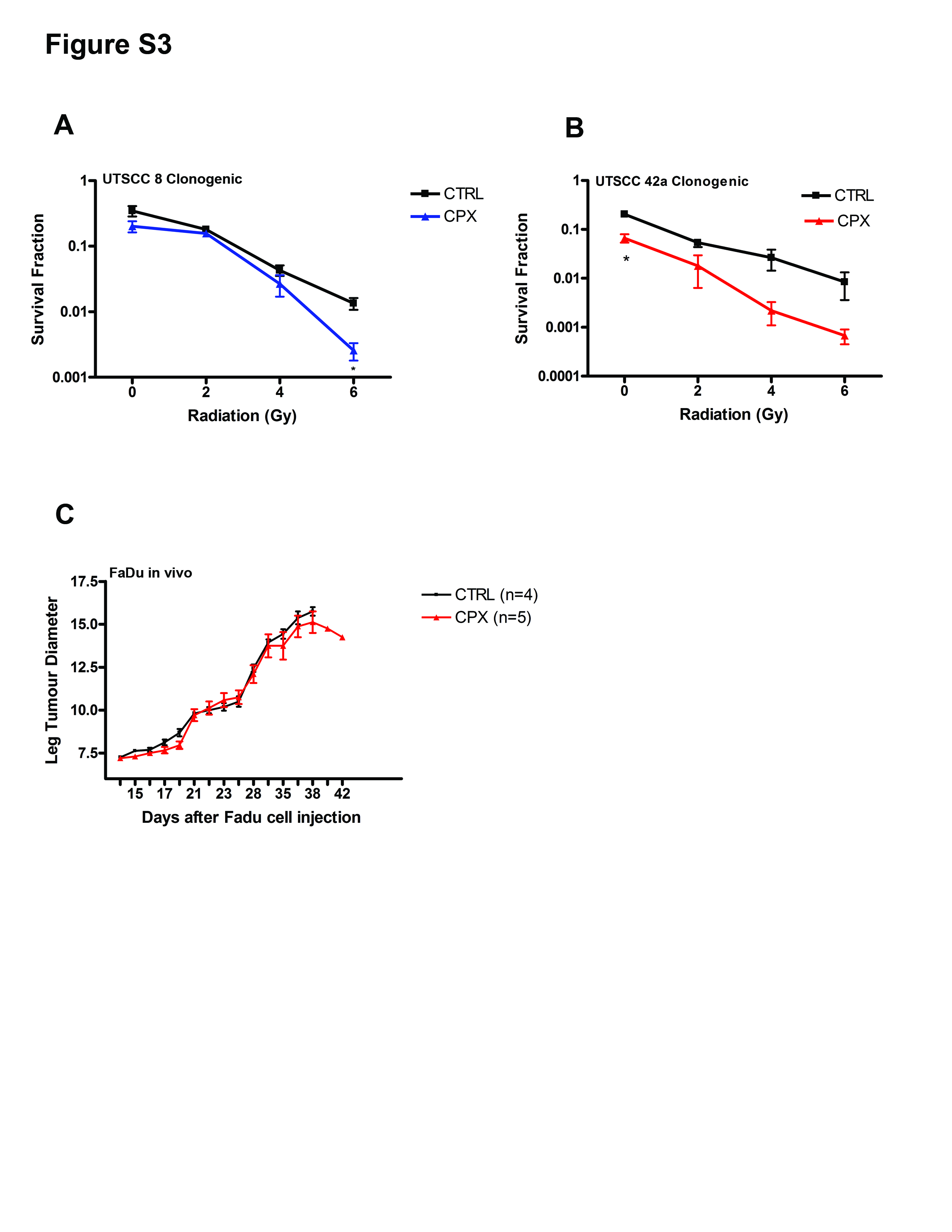

Supplement: Figure S3 — Ciclopirox olamine reduced HNSCC clonogenicity. (A) Clonogenic survival of UTSCC8 cells was measured 10 to 12 days after re-seeding of cells treated with ethanol (5 uM) or CPX (5 uM) and RT (0, 2, 4 or 6 Gy) 72 hours after CPX treatment. (B) Clonogenic survival of UTSCC42a cells was measured 10 to 12 days after re-seeding cells treated with ethanol (5 uM) or CPX (5 uM) and RT (0, 2, 4 or 6 Gy) 72 hours after CPX treatment. (C) FaDu tumors were established in SCID mice; once TLD reached ~8 mm, mice were randomly assigned to vehicle (water) or CPX, administered as oral dosages (25 mg/kg) five times per week for a total of 2 weeks. Each treatment group comprised of at least four mice. *P<0.05 P=ns (not significant). (TIF) [file pone.0074075.s003.tif]

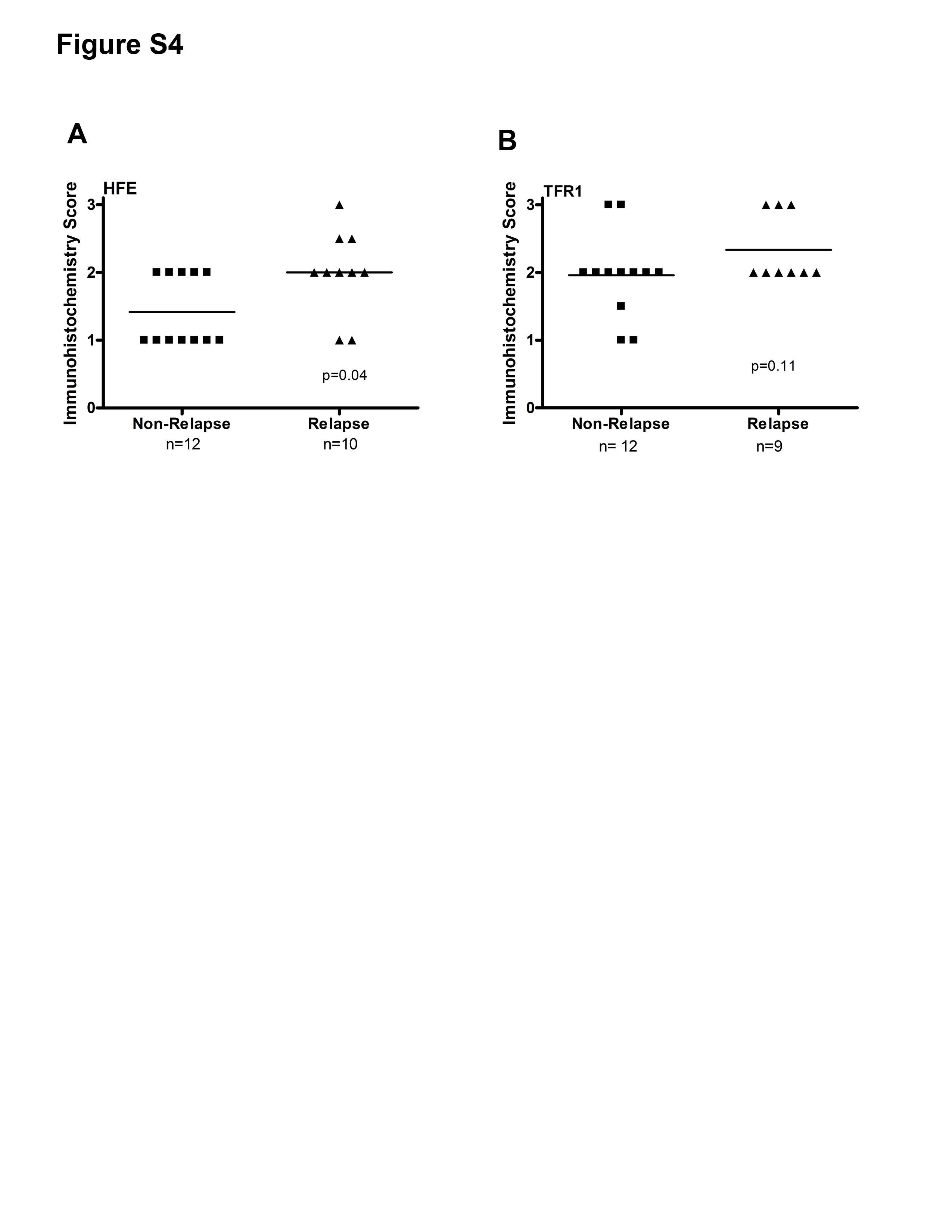

Supplement: Figure S4 — HFE and TFR1 IHC immune-expression score between non-relapsed vs. relapsed HNSCC patient samples. (A) HFE immunohistochemistry score (1–3), for non-relapsed (n=12) vs. relapsed (n=10) HNSCC patient samples. (B) TFR1 immunohistochemistry score (1–3) for non-relapsed (n=12) vs. relapsed (n=9) HNSCC patient samples. (TIF) [file pone.0074075.s004.tif]

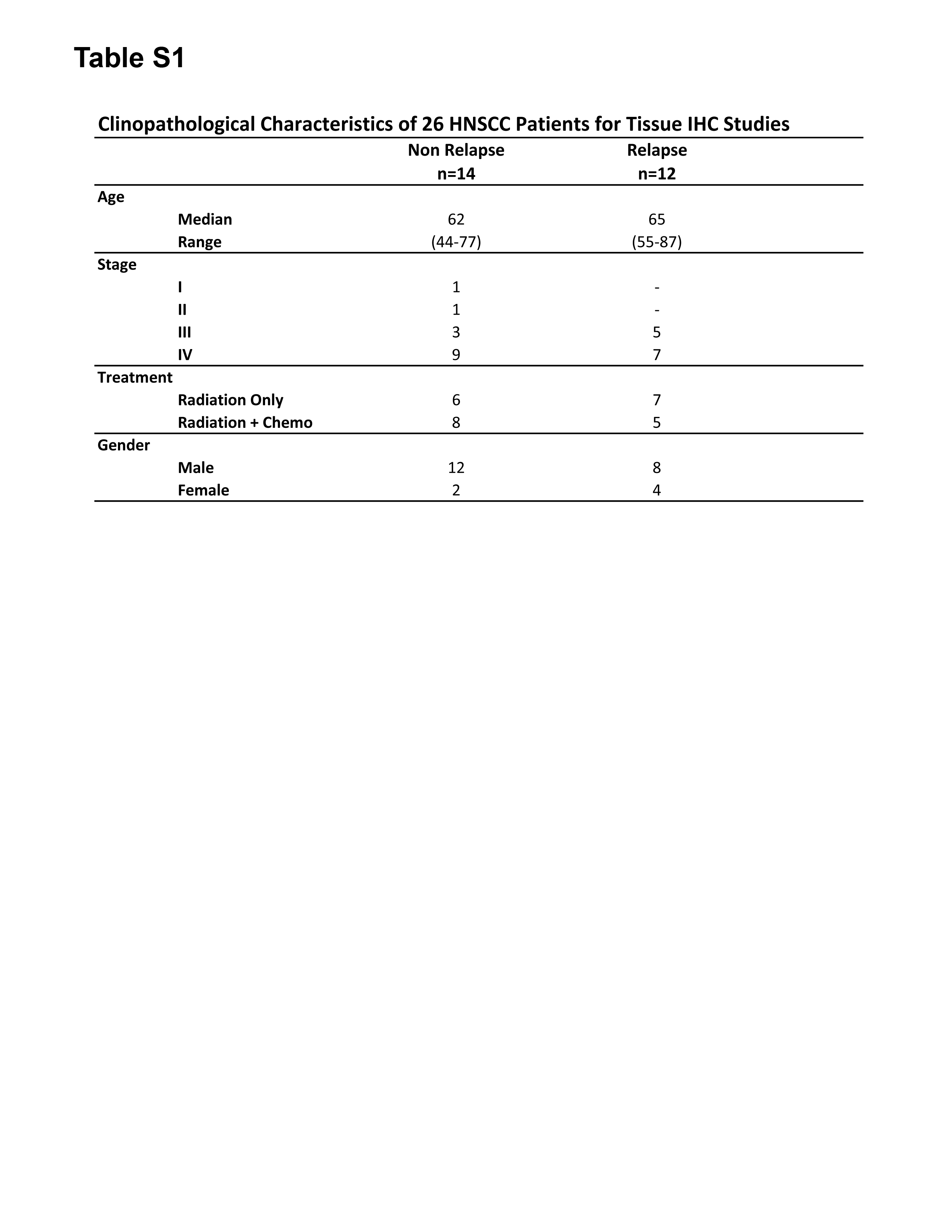

Supplement: Table S1 — Clinical details of the 26 HNSCC patients. (TIF) [file pone.0074075.s005.tif]

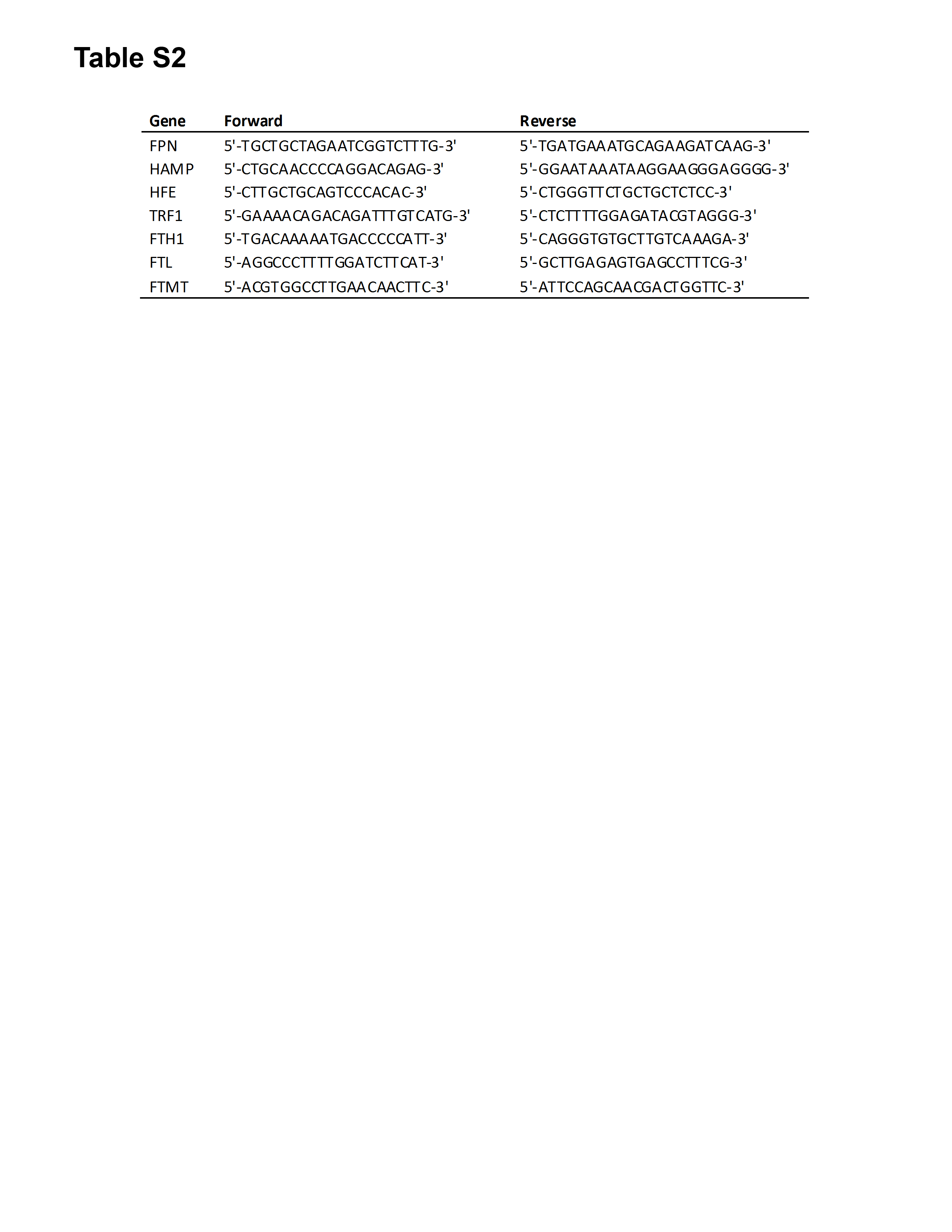

Supplement: Table S2 — qRT-PCR primer design sequences. (TIF) [file pone.0074075.s006.tif]
